# The association between medication or alcohol use and the incidence of frailty: a retrospective cohort study

**DOI:** 10.1186/s12877-020-01969-y

**Published:** 2021-01-07

**Authors:** Janja Jazbar, Igor Locatelli, Mitja Kos

**Affiliations:** University of Ljubljana, Faculty of Pharmacy, Askerceva cesta 7, 1000 Ljubljana, Slovenia

**Keywords:** Frailty index, Frailty phenotype, Longitudinal study, SHARE data, Medication use, Alcohol use

## Abstract

**Background:**

Understanding potentially modifiable factors that influence the risk of frailty is a key concern for the management of this urgent contemporary public health challenge. This study evaluates the association between the use of various medications or alcohol and the incidence of frailty among older adults.

**Methods:**

This study was a retrospective cohort study on older adults (≥ 65 years) using data from the longitudinal Survey of Health, Ageing and Retirement in Europe (SHARE survey, 28 countries). Medication use was measured as taking several different groups of medications. Alcohol use was assessed with SHARE questions corresponding to AUDIT-C. The outcome measure was the incidence of frailty after two years, defined by frailty index (FI) and frailty phenotype (FP). A multiple logistic regression model was used to evaluate the association with adjustment for several potential confounding factors.

**Results:**

Of the 14,665 FI-population participants, 1800 (12.3%) developed frailty within two years. Of the 8133 FP-population participants, 2798 (34.4%) developed pre-frailty and 247 (3.0%) developed frailty within two years of baseline. After adjustment for potential confounding variables, non-hazardous alcohol use (adjusted OR; 95% CI for the FI-population: 0.68; 0.60–0.77) and hazardous alcohol use (0.80; 0.68–0.93) are associated with lower incidence of frailty compared to no alcohol use. The odds of frailty are increased when taking medications; the largest effect size was observed in older adults taking medication for chronic bronchitis (adjusted OR; 95% CI for the FI-population: 2.45; 1.87–3.22), joint pain and other pain medication (2.26; 2.00–2.54), medication for coronary and other heart disease (1.72; 1.52–1.96), medication for diabetes (1.69; 1.46–1.96), and medication for anxiety, depression and sleep problems (1.56; 1.33–1.84). Additionally, the risk of frailty was increased with stroke, Parkinson’s disease and dementia.

**Conclusions:**

Taking certain groups of medication was associated with increased incidence of frailty and pre-frailty, which might be due to either medication use or the underlying disease. Alcohol use was associated with a lower risk of pre-frailty and frailty compared to no alcohol use, which might be due to reverse causality or residual confounding. There was no significant interaction effect between medication groups and alcohol use on frailty incidence.

**Supplementary Information:**

The online version contains supplementary material available at 10.1186/s12877-020-01969-y.

## Background

Frailty, a syndrome that is associated with old age, is among the most urgent contemporary public health challenges due to global trends of population aging [1]. Frailty can be characterized as a decline in the physiological capacity of multiple organ systems, leading to increased vulnerability to stressor events [1–3]. It is also associated with an increased risk of falls, fractures, hospitalization, institutionalization, impaired quality of life and mortality [2]. How to objectively measure frailty is extensively debated in the medical literature, with two main methods being widely recognized. The first method, ‘frailty phenotype’, defines frailty as the presence of at least three out of the following five physical components: shrinking, weakness, exhaustion, slowness and low levels of physical activity. Pre-frailty is defined as the presence of one or two of these five physical components [2, 4]. The second method defines a ‘frailty index’ as the accumulation of age-related deficits, taken from over thirty health deficits that include comorbidities, signs, symptoms, physiological factors and disabilities [2, 5].

Although frailty prevalence increases with age, it is not an inevitable result of aging [2]. Therefore, understanding potentially modifiable factors that influence the risk of frailty is a key concern for its management. The association between alcohol use and incident frailty remains controversial, with only four studies included in a recent meta-analysis [6]. Short-term studies suggest lower incidence of frailty with higher alcohol use [6], while recent long-term study reported higher incidence of frailty in old age with high alcohol use in mid-life [7]. Also, different disease or accompanying medication use have been associated with increased frailty, for example multiple sclerosis, diabetes, chronic obstructive pulmonary disease or taking antidepressant or psychotropic medications [8–11]. Nevertheless, no study so far comprehensively evaluated the risk of frailty in patients taking different pharmacotherapy groups of medication. Furthermore, alcohol has the potential to interact with medications, with possible harmful health outcomes, for example sedation, hypotension, hypoglycaemia or gastrointestinal bleeding [12]. The possible influence of this interaction on frailty has not yet been investigated.

The aim of our study was to evaluate the association between use of different medications or alcohol and the incidence of frailty in order to specify groups of older adults with higher risk of becoming frail.

## Methods

### Study design

This retrospective cohort study on older adults uses data from the longitudinal Survey of Health, Ageing and Retirement in Europe (SHARE survey). Data from 2013 (Wave 5) and 2015 (Wave 6) are used for the analysis [13, 14]. Study exposures are disease, medication and alcohol use measured at baseline (2013, Wave 5). The outcome measure of the study is the incidence of frailty syndrome two years after baseline (2015, Wave 6).

### Study data

The SHARE survey is a large-scale longitudinal and multidisciplinary study of over 140,000 individuals, aged 50 years and older, from 27 European countries and Israel (the SHARE survey countries). The first wave began in 2004 (Wave 1), and new waves are released approximately every two years; the most recent data from regular questionnaires were used in the current study (Waves 5 and 6). Detailed methodologies used in the SHARE survey are described elsewhere [15–18]. The SHARE survey is subject to continuous ethics review; Waves 5 and 6 were reviewed and approved by the Ethics Council of the Max Planck Society [19].

### Study population

SHARE survey participants are aged 50 years and over at the time of sampling, and they all live in one of the SHARE survey countries. The inclusion criteria for the current study were: (i) participants in both 2013 and 2015 SHARE survey, and (ii) aged 65 years or more in 2013. This study population was formed of 25,225 individuals with a mean (SD) age of 73.8 (6.6) years, and of whom 55.5% were female and 44.5% were male. The study participants lived in the following countries: Austria, Germany, Sweden, Spain, Italy, France, Denmark, Switzerland, Belgium, Israel, Czech Republic, Luxembourg, Slovenia and Estonia. The final study analysis was restricted to participants who were classified as non-frail (using the two described frailty measures) at baseline (Wave 5, 2013) and who had complete datasets for all study variables at both the 2013 and 2015 timepoints. Thus, the final study analysis was performed on data from: (i) 14,665 participants who were classified as non-frail using the ‘frailty index’, and (ii) 8133 individuals who were classified as non-frail using the ‘frailty phenotype’. The difference in absolute number of non-frail participants according to each method is the consequence of three frailty categories according to ‘frailty phenotype’ (non-frail, pre-frail, frail) and two frailty categories according to ‘frailty index’ (non-frail, frail).

### Frailty measures

The outcome measure in this study was frailty syndrome, as defined by two validated methods: frailty index and frailty phenotype.

*Frailty phenotype (FP),* first defined in 2001 by Fried et al., defines frailty syndrome as the presence of three or more of five pre-defined physical components: shrinking, weakness, exhaustion, slowness and low levels of physical activity [4]. We use the previously published operationalization of frailty phenotype (FP) on SHARE data in our study; details are presented in Additional file 1 [20–23]. This method has been validated in relation to different health outcomes, although we acknowledge the differences from the original method [20, 21]. Study participants were classified into the following groupings: (i) non-frail (robust) if none of the components were present; (ii) pre-frail if one or two of the components were present; and (iii) frail if three to five components were present.

*Frailty index (FI),* first defined in 2001 by Mitnitski et al., defines frailty syndrome through an accumulation of age-related deficits, which are typically taken from a list of at least thirty deficits [5]. Frailty index (FI) has also been defined previously using SHARE survey data from Waves 1 and 2 (‘SHARE-FI’) [21, 24]. In the current study, we adjusted the previously published SHARE-FI for use in data from Waves 5 and 6, in accordance with the guidelines published by Searle et al. [25] The age-related deficits from which the FI is calculated are listed in Additional file 2. In accordance with previous studies, participants with FI scores ≥0.25 were considered to be frail [21, 26].

### Exposure measures

Exposure to medications and the underlaying disease was evaluated through the question “Do you currently take drugs at least once a week for (health) problems mentioned on this card?”, with the following medication groups: (i) high blood cholesterol, (ii) high blood pressure, (iii) coronary or cerebrovascular diseases and other heart diseases, (iv) diabetes, (v) joint pain or joint inflammation and other pain (e.g., headache, back pain, etc.), (vi) sleep problems, anxiety or depression, (vii) osteoporosis, (viii) stomach burns, (ix) chronic bronchitis, and (x) drugs for inflammation suppression (only glucocorticoids or steroids). Additionally, health status was further characterized by the following diseases not included in the medication list: stroke, cancer, Parkinson’s disease, dementia (Alzheimer disease, dementia or other serious memory impairment), and cataracts; these were assessed using the following question: “Has a doctor ever told you that you had/Do you currently have any of the conditions on this card?”

Baseline alcohol use was assessed from SHARE survey data that were adapted to their equivalent in the AUDIT-C test. The following questions were used: (i) “During the last 3 months, how often have you drunk any alcoholic beverages, like beer, cider, wine, spirits or cocktails?”, (ii) “In the last three months, on the days you drank , about how many drinks do you have?”, (iii) “In the last three months, how often did you have six or more drinks on one occasion?”. Answers to each question were categorized into 5 categories from 0 to 4 points. For each participant a total sum for all three questions was calculated and then categorized as follows: (i) no alcohol use (0 points), (ii) non-hazardous alcohol use (1–3 points in women and 1–4 points in men), or (iii) hazardous alcohol use (≥4 point in women and ≥ 5 points in men) [27]. Detailed method was published and validated previously [27–29].

### Covariates

The study model included the following potential confounding factors: age, sex, country of residence, highest educational level, BMI and smoking (yes/no). Age was categorized as follows: (i) 65–74 years, (ii) 75–84 years, and (iii) ≥85 years. Highest educational level was categorized based on ISCED 1997 coding into three categories: primary, secondary, tertiary. BMI was categorized into four groups: (i) < 18.5, (ii) 18.5–25, (iii) 25–30, and (iv) > 30.

### Statistical analysis

Multiple logistic regression model was used to evaluate the influence of medication and alcohol exposure at the 2013 baseline on the incidence of frailty syndrome two years later, in 2015. The two frailty measures, FI and FP, were used as the outcome measures in two separate analyses. All exposure variables and potential confounding variables were entered in the model concomitantly: alcohol use, medication use, stroke, cancer, Parkinson’s disease, dementia, cataracts, age, sex, country of residence, BMI, highest educational level and smoking. Separate analyses were performed with the addition of the interaction effect between alcohol use and each specific medication group in the model.

## Results

### Study population characteristics

The final study populations, after excluding participants that had missing data, were 14,665 based on the FI (FI-population) and 8133 based on FP (FP-population); all the final study participants were considered to be non-frail at baseline. Basic characteristics of both study populations are presented in Table 1. The mean ages (SD) were 72.5 (6.0) years (FI-population) and 71.6 (5.4) years (FP-population). More than half of the study participants were non-hazardous alcohol users at baseline, with approximately one fifth being hazardous alcohol users (22.4 and 23.3% for the FI- and FP-populations, respectively). No alcohol use at baseline was reported by 26.7% of the FI-population and 22.4% of the FP-population. At baseline, almost half of the study participants were taking medications for high blood pressure and approximately 1 in 4 had medications for high blood cholesterol. Between 10 and 20% of the participants were taking medications for coronary and other heart disease, joint pain and other pain, and diabetes. The prevalence of other medication groups was less than 10% among non-frail baseline participants.

**Table 1:** Baseline characteristics of the study population. (FI-population *N* = 14,665; FP-population *N* = 8133)

Baseline characteristic	Non-frail population at baseline based on frailty index (FI-population)	Non-frail population at baseline based on frailty phenotype (FP-population)
	N = 14,665	N = 8133
Age group		
65–74 years	9870 (67.3%)	5979 (73.5%)
75–84 years	4184 (28.5%)	1948 (24.0%)
≥ 85 years	611 (4.2%)	206 (2.5%)
Mean (SD)	72.5 (6.0)	71.6 (5.4)
Sex		
Male	7312 (49.9%)	4244 (52.2%)
Female	7353 (50.1%)	3889 (47.8%)
Highest educational level		
primary	3258 (22.2%)	1584 (19.5%)
secundary	7900 (53.9%)	4441 (54.6%)
tertiary	3507 (23.9%)	2108 (25.9%)
Body mass index		
under 18.5	149 (1.0%)	69 (0.8%)
18.5–25	5379 (36.7%)	3053 (37.5%)
25–30	6480 (44.2%)	3668 (45.1%)
above 30	2657 (18.1%)	1343 (16.5%)
No. of chronic diseases; Mean (SD)	1.7 (1.3)	1.5 (1.3)
Smoking (yes)	2120 (14.5%)	1129 (13.9%)
Alcohol use		
no alcohol use	3915 (26.7%)	1823 (22.4%)
nonhazardous	7472 (51.0%)	4413 (54.3%)
hazardous	3278 (22.4%)	1897 (23.3%)
Stroke (yes)	392 (2.7%)	188 (2.3%)
Cancer (yes)	694 (4.7%)	378 (4.6%)
Parkinson disease (yes)	72 (0.5%)	34 (0.4%)
Cataracts (yes)	1487 (10.1%)	820 (10.1%)
Dementia (yes)	75 (0.5%)	31 (0.4%)
Medication for high blood pressure (yes)	7090 (48.3%)	3830 (47.1%)
Medication for coronary and other heart disease (yes)	2580 (17.6%)	1237 (15.2%)
Medication for high cholesterol (yes)	3908 (26.6%)	2180 (26.8%)
Medication for diabetes (yes)	1646 (11.2%)	877 (10.8%)
Medication for joint pain and other pain (yes)	2645 (18.0%)	1125 (13.8%)
Medication for anxiety, depression and sleep problems (yes)	1248 (8.5%)	521 (6.4%)
Medication for osteoporosis (yes)	777 (5.3%)	378 (4.6%)
Medication for stomach burns (yes)	1082 (7.4%)	522 (6.4%)
Medication for chronic bronchitis (yes)	347 (2.4%)	140 (1.7%)
Medication for suppresing inflammation (yes)	384 (2.6%)	163 (2.0%)

### Frailty incidence

Of the 14,665 FI-population participants, 1800 (12.3%) developed frailty within two years of baseline; the incidence of frailty increased considerably with baseline age and was higher among females. The incidence of frailty in the population that reported no baseline alcohol use was 18.8%, whereas the incidence was lower in non-hazardous (9.9%) and hazardous (10.0%) alcohol users. FI-population participants with medications had a higher incidence of frailty compared to participants without specific medication group (Table 2). The highest incidence of frailty was reported in the participants taking medications for chronic bronchitis (25.1%), joint pain and other pain medications (22.8%) and medications for anxiety, depression and sleep problems (20.9%).

**Table 2:** Association between alcohol use, medication use and frailty incidence in the multiple model

Variable	Frailty index – frail		Frailty phenotype – pre-frail		Frailty phenotype – frail	
	Incidence of frailty (FI)	Adjusted OR^a^ (95% CI)	Incidence of pre-frailty (FP)	Adjusted OR^a^ (95% CI)	Incidence of frailty (FP)	Adjusted OR^a^ (95% CI)
Age						
65–74 years	8.7%	Reference	31.4%	Reference	2.0%	Reference
75–84 years	18.0%	2.10 (1.87–2.36)**	41.6%	1.57 (1.40–1.76)**	4.8%	2.33 (1.71–3.17)**
≥ 85 years	30.4%	5.23 (4.25–6.45)**	51.9%	3.46 (2.50–4.78)**	16.0%	17.0 (10.1–28.7)**
Sex						
Male	10.9%	Reference	32.1%	Reference	3.0%	Reference
Female	13.7%	1.18 (1.05–1.33)*	37.0%	1.19 (1.08–1.32)**	3.1%	1.07 (0.79–1.45)
Alcohol use						
No alcohol use	18.8%	Reference	40.9%	Reference	4.7%	Reference
Non-hazardous	9.9%	0.68 (0.60–0.77)**	32.3%	0.86 (0.76–0.98)*	2.4%	0.66 (0.47–0.93)*
Hazardous	10.0%	0.80 (0.68–0.93)*	32.9%	0.93 (0.80–1.08)	2.8%	0.88 (0.58–1.32)
Stroke						
No	11.9%	Reference	34.3%	Reference	3.0%	Reference
Yes	24.2%	2.10 (1.61–2.73)**	40.4%	1.22 (0.88–1.68)	5.9%	1.82 (0.89–3.74)
Cancer						
No	12.2%	Reference	34.4%	Reference	3.1%	Reference
Yes	13.0%	1.23 (0.96–1.57)	34,1%	1.01 (0.80–1.27)	2.6%	0.98 (0.49–1.97)
Parkinson’s disease						
No	12.2%	Reference	34.4%	Reference	3.0%	Reference
Yes	34.7%	4.29 (2.48–7.43)**	41.2%	1.80 (0.83–3.94)	17.6%	7.58 (2.47–23.2)**
Cataracts						
No	11.9%	Reference	34.0%	Reference	2.7%	Reference
Yes	15.2%	1.12 (0.95–1.33)	37.7%	1.13 (0.96–1.33)	6.2%	2.07 (1.43–3.01)**
Dementia						
No	12.1%	Reference	34.4%	Reference	3.0%	Reference
Yes	38.7%	3.97 (2.34–6.72)**	45.2%	1.45 (0.68–3.10)	3.2%	0.59 (0.07–5.16)
Med. for high blood pressure						
No	9.6%	Reference	31.9%	Reference	2.3%	Reference
Yes	15.2%	1.35 (1.21–1.51)**	37.3%	1.15 (1.04–1.28)*	3.8%	1.37 (1.02–1.84)*
Med. for coronary and other heart disease						
No	10.6%	Reference	33.1%	Reference	2.6%	Reference
Yes	20.1%	1.72 (1.52–1.96)**	41.4%	1.28 (1.12–1.47)**	5.3%	1.70 (1.20–2.41)*
Med. for high blood cholesterol						
No	11.5%	Reference	33.9%	Reference	3.1%	Reference
Yes	14.3%	1.08 (0.96–1.22)	35.6%	0.93 (0.83–1.05)	3.0%	0.68 (0.49–0.95)*
Med. for diabetes						
No	11.5%	Reference	34.1%	Reference	2.8%	Reference
Yes	18.6%	1.69 (1.46–1.96)**	37.1%	1.11 (0.95–1.30)	5.2%	1.62 (1.11–2.37)*
Med. for joint and other pain						
No	10.0%	Reference	32.6%	Reference	2.6%	Reference
Yes	22.8%	2.26 (2.00–2.54)**	45.9%	1.58 (1.38–1.82)**	6.0%	2.38 (1.70–3.34)**
Med. for anxiety, depression and sleep problems						
No	11.5%	Reference	33.5%	Reference	2.8%	Reference
Yes	20.9%	1.56 (1.33–1.84)**	47.4%	1.56 (1.28–1.89)**	6.3%	2.14 (1.37–3.36)*
Med. for osteoporosis						
No	11.9%	Reference	34.0%	Reference	3.0%	Reference
Yes	19.2%	1.56 (1.27–1.93)**	43.4%	1.38 (1.10–1.73)*	4.2%	1.34 (0.74–2.45)
Med. for stomach burns						
No	11.9%	Reference	34.0%	Reference	3.0%	Reference
Yes	16.5%	1.14 (0.94–1.37)	40.0%	1.11 (0.91–1.35)	3.4%	1.01 (0.58–1.74)
Med. for chronic bronchitis						
No	12.0%	Reference	34.2%	Reference	3.0%	Reference
Yes	25.1%	2.45 (1.87–3.22)**	47.1%	1.68 (1.17–2.42)*	6.4%	2.67 (1.20–5.95)*
Med. for inflammation						
No	12.1%	Reference	34.2%	Reference	3.0%	Reference
Yes	17.4%	1.54 (1.14–2.05)*	44.2%	1.32 (0.94–1.85)	4.9%	1.61 (0.70–3.66)

*Note*. *OR* Odds ratio, *CI* Confidence interval

^a^Variables used in the multivariate model: country of residence, BMI, highest educational level, smoking, and variables presented in the table. Omnibus test p < 0.001 (FI and FP models). Nagelkerke R square = 0.196 (frailty FI); Nagelkerke R square = 0.089 (pre-frailty FP); Nagelkerke R square = 0.223 (frailty FP). N = 14,665 for FI; N = 8133 for FP

*p < 0.05

**p < 0.001

Of the 8133 FP-population participants, 2798 (34.4%) developed pre-frailty and 247 (3.0%) developed frailty within two years of baseline. The incidences of pre-frailty and frailty in the population that reported no baseline alcohol use were 40.9 and 4.7%, respectively. The incidences were similarly lower in non-hazardous and hazardous alcohol users (32.3% for pre-frailty in non-hazardous and 32.9% in hazardous alcohol users, and 2.4% for frailty in non-hazardous and 2.8% for frailty in hazardous alcohol users). Similar to the FI-population, older adults taking medications had higher incidences of pre-frailty and frailty, with the highest reported for medications for chronic bronchitis, joint pain and other pain medications and medications for anxiety, depression and sleep problems (Table 2). Only older adults taking medications for high blood cholesterol had slightly lower incidence of frailty (3.0% compared to 3.1% in the population without these medications).

### Association between medication or baseline alcohol use, and frailty incidence

After adjustment for potential confounding variables, both medication and baseline alcohol use show a statistically significant association with frailty incidence when measured by the FI, with odds ratios (95% CI) presented in Table 2. Both non-hazardous and hazardous alcohol use are associated with lower odds of frailty incidence compared to no alcohol use. The odds for frailty are increased when taking medications, with significant difference (adjusted OR, 95% CI) for medications for high blood pressure (1.35, 1.21–1.51), coronary and other heart disease (1.72, 1.52–1.96), diabetes (1.69, 1.46–1.96), joint pain and other pain (2.26, 2.00–2.54), anxiety, depression and sleep problems (1.56, 1.33–1.84), osteoporosis (1.56, 1.27–1.93), chronic bronchitis (2.45, 1.87–3.22) and suppressing inflammation (1.54, 1.14–2.05).

In case of FP (Table 2), non-hazardous alcohol use is significantly associated with lower odds of pre-frailty and frailty compared to no alcohol use (adjusted OR, 95% CI: 0.86, 0.76–0.98 for pre-frailty and 0.66, 0.47–0.93 for frailty). Hazardous alcohol use shows no significant difference compared to no alcohol use. Significantly increased odds for pre-frailty and frailty were confirmed for the following medication groups: high blood pressure (1.15, 1.04–1.28 for pre-frailty and 1.37, 1.02–1.84 for frailty), coronary and other heart disease (1.28, 1.12–1.47 for pre-frailty and 1.70, 1.20–2.41 for frailty), joint pain and other pain (1.58, 1.38–1.82 for pre-frailty and 2.38, 1.70–3.34 for frailty), anxiety, depression and sleep problems (1.56, 1.28–1.89 for pre-frailty and 2.14, 1.37–3.36 for frailty), and chronic bronchitis (1.68, 1.17–2.42 for pre-frailty and 2.67, 1.20–5.95 for frailty). Additionally, taking medications for diabetes was significantly associated with frailty (1.62, 1.11–2.37) and taking medications for osteoporosis was significantly associated with pre-frailty (1.38, 1.1–1.73). Taking medications for high blood cholesterol was significantly associated with lower odds for frailty (0.68, 0.49–0.95).

The interaction between each medication group and baseline alcohol use shows no statistically significant influence on frailty syndrome for either the FI-population or the FP-population (multiple logistic regression model with interaction terms; data not shown).

## Discussion

The present study explores the complex relationship between medication and alcohol use and the incidence of frailty in a large cohort of older adults. The incidence of frailty in our study was increased in participants with various groups of medication and in participants with no alcohol use. There was no effect of interaction between medication and alcohol use on the incidence of frailty.

Older adults taking certain medications had increased risk of frailty and pre-frailty in our study, indicating which groups of older adults are at higher risk of developing frailty, either due to adverse effects of medication use or the underlying disease. The greatest effect size was observed in older adults taking medications for chronic bronchitis, medications for joint pain and other pain, medications for coronary and other heart disease, medications for diabetes, and medications for anxiety, depression and sleep problems in both FI- and FP-population. Among disease states, significant association with increased incident frailty was found in older adults with stroke (FI-population), Parkinson’s disease (FI- and FP-population), dementia (FI-population), and cataracts (FP-population). Our findings are consistent with study published in Lancet Public Health [30], which also reported high association of frailty with chronic obstructive pulmonary disease, diabetes, stroke, coronary heart disease, depression and pain among other long-term conditions. Our study confirms relationship between cognitive health and frailty, since older adults with medication for pain, anxiety, depression and sleep problems were at significantly increased risk of frailty. Although the theory of this association remains poorly explored, relationship between cognition and frailty was also confirmed in a systematic review [31]. Furthermore, certain central nervous system medications are related to falls [32], which might be either antecedents or manifestations of frailty [33]. Interestingly, in the FP-population reduced incidence of frailty was found in older adults taking high blood cholesterol medications. Due to the asymptomatic nature of hyperholesterolaemia, association to increased risk of frailty might be less expected, unless it was related to secondary cardiovascular outcomes. On the other hand some studies have shown association between statin use and improved physical performance [10]. Nevertheless, a prospective study of statin use and incident frailty in women showed no association [10]. The results of our study could also be explained by residual confounding not included in our model, therefore further studies are needed to explore this finding.

The effect of medications cannot be separated from the effect of the underlying disease in our study. The correlation between disease and corresponding medication use in our data was too high to include both variables in the model. Our study results thus indicate which groups of older adults are at higher risk of frailty and should be monitored more closely, but the causal mechanisms of frailty remain to be explored.

The influence of alcohol consumption on frailty risk is controversial. A few longitudinal studies show an inverse correlation between alcohol consumption and frailty risk [6, 34–37]. The results of the current study have similar implications. Non-hazardous and hazardous alcohol use in old age showed decreased frailty risk compared to no alcohol use, even after adjustment for baseline disease and medication use; however, hazardous alcohol use showed smaller decrease than non-hazardous alcohol use (adjusted OR (95% CI): 0.68 (0.60–0.77) for non-hazardous use and 0.80 (0.68–0.93) for hazardous alcohol use). Similarly, when using frailty phenotype as the outcome measure, only non-hazardous alcohol use was associated with decreased frailty incidence, suggesting a U-shaped relationship, which has been proposed before [6, 38, 39]. Sub-analysis of our data using previous alcohol problems as additional cofactor showed that participants with previous alcohol problems (self-reported excessive drinking problem in the past) have significantly increased incidence of frailty compared to participants without previous alcohol problems, even after adjustment for several confounding factors. This variable was not included in our main analysis because only a subpopulation of participants with current alcohol use had to fulfill this question. This sub-analysis result is in accordance with a 30-year follow-up study published by Strandberg et al. [7] where alcohol use was shown to be an independent risk factor for developing frailty in old age among men who had high alcohol consumption during mid-life [7]. Previous authors have suggested that alcohol use in old age might be the result of reverse causality, whereby older adults with health problems choose to limit or reduce their alcohol use even before the presentation of frailty [6, 7, 40]. Death before old age is another important consideration influencing the relationship between alcohol use and frailty risk in short-term follow-up studies on older adults [6, 7]. Furthermore, Kojima et al. found that high association between nondrinkers and frailty might be explained by poorer baseline health status [40]. Although the model in our study was adjusted for several baseline health status conditions, there is still possibility of residual confounding. Future studies with long-term follow-up and adjustment for various potential health-related confounding factor are warranted.

The effect of interaction between medication and alcohol use showed no significant association with frailty incidence in our study. This interaction effect is more likely associated with very specific alcohol interacting medications rather than a group of medications [41]. Further studies are needed to explore the effects of such specific alcohol-medication interactions on the risk of frailty in older adults.

The incidence of frailty in our study was 12.3% in the FI-population and 3.0% in the FP-population, during a two-year follow-up period. Additionally, incidence of prefrailty in the FP-population was 34.4%. The difference between the two measures is considerable but consistent with published literature [39, 40]. The meta-analysis on frailty incidence reported 4.6% pooled incidence of frailty among robust older adults aged 60 years and older during a median follow-up of three years. Great majority of studies included in the meta-analysis measured frailty with frailty phenotype (FP). Studies using frailty index (FI) as diagnostic measure typically reported higher incidence of frailty. We acknowledge the differences between FP and FI concept and recognize both methods as important and leading methods for defining frailty [2, 42]. Therefore, we included both methods as an outcome in our study.

The study was conducted on a large population of older adults. The Survey of Health, Ageing and Retirement in Europe (SHARE) covers multiple European countries and uses objective and validated health measures. Frailty was assessed with two widely recognized methods and baseline alcohol use was evaluated using the validated AUDIT-C questionnaire. The main limitation of our study is lack of detailed data on medication use, which would enable further study of alcohol-medication interaction and the influence of specific medications on frailty risk. Due to the multidisciplinary nature of the SHARE survey, several confounding factors were adjusted for; nevertheless, potential residual confounding factors should be considered when interpreting the study results.

## Conclusion

The present study explores the complex relationship between medication and alcohol use, and the incidence of frailty in older adults. Taking certain groups of medication was associated with increased incidence of frailty and pre-frailty, which might be due to either adverse effects of medication use or the underlying disease. The greatest effect size was observed in older adults taking medications for chronic bronchitis, joint pain and other pain medications, medications for coronary and other heart disease, medications for diabetes, and medications for anxiety, depression and sleep problems. In addition, association with frailty was found in older adults diagnosed with stroke, Parkinson’s disease and dementia. Non-hazardous alcohol use was associated with a lower risk of frailty and pre-frailty compared to no alcohol use. Older adults with hazardous alcohol use were not significantly different from older adults with no alcohol use when using frailty phenotype as the outcome measure. The reversed association between alcohol use in old age and frailty risk might be due to reverse causality or residual confounding. The effect of interaction between medication and alcohol use showed no significant association with frailty incidence in our study.

This study informs health professionals which groups of non-frail older adults have a higher probability of progressing to frailty. In addition to reducing risk of frailty with exercise and nutrition, concerted efforts need to be made to optimize the medical condition and minimize adverse effects of medication therapy.

## Supplementary Information

**Additional file 1: Table A.** Variables used to define Frailty phenotype (FP) from SHARE data.

**Additional file 2: Table B.** Variables used to define Frailty index from SHARE data.

## Data Availability

Access to the data collected and generated in the SHARE projects is provided free of charge for scientific use globally, subject to European Union and national data protection laws as well as the publicly available Conditions of Use. Access to the data is available online (http://www.share-project.org/data-access.html) after user registration.

## Abbreviations

FI: Frailty index
FP: Frailty phenotype
SHARE: Survey of health, aging and retirement in Europe
BMI: Body mass index
ISCED: International standard classification of education
SD: Standard deviation
OR: Odds ratio
CI: Confidence interval
AUDIT: The alcohol use disorders identification test
