# Supplementary material for: The association between medication or alcohol use and the incidence of frailty: a retrospective cohort study
Source: BMC Geriatr. 2021 Jan 7;21:25. doi: 10.1186/s12877-020-01969-y (PMC7791729; doi:10.1186/s12877-020-01969-y)
Supplement: Supplementary file 1 — Additional file 1: Table A. Variables used to define Frailty phenotype (FP) from SHARE data. [file 12877_2020_1969_MOESM1_ESM.docx]

***Additional file 1. Frailty phenotype (FP) defined from SHARE data***

Study participants were classified into the following groupings: (i) non-frail (robust) if none of the components were present; (ii) pre-frail if one or two of the components were present; and (iii) frail if three to five components were present.

**Table A. Variables used to define Frailty phenotype (FP) from SHARE data.**

| **Frailty component** | **Frailty phenotype defined by SHARE** |
| --- | --- |
| **Shrinking** | What has your appetite been like in the last month?  No diminution in desire for food = 0; Diminution in desire for food = 1  If an uncodable response: “So, have you been eating more or less than usual?”  More = 0; Neither more nor less = 0; Less = 1 |
| **Weakness** | Handgrip strength measured by dynamometer  Men  BMI ≤ 24 and strength ≤ 29 = 1  BMI 24.1–26 and strength ≤ 30 = 1  BMI 26.1–28 and strength ≤ 30 = 1  BMI > 28 and strength ≤ 32 = 1  Women  BMI ≤ 23 and strength ≤ 17 = 1  BMI 23.1–26 and strength ≤ 17.3 = 1  BMI 26.1–29 and strength ≤ 18 = 1  BMI > 29 and strength ≤ 21 = 1 |
| **Exhaustion** | In the last month, have you had too little energy to do the things you wanted to do?  No = 0; Yes = 1 |
| **Slowness** | Please tell me whether you have any difficulty [expected to last more than 3 months] walking 100 m or … climbing one flight of stairs without resting.  No difficulties = 0; Difficulty with either task = 1 |
| **Low levels of physical activity** | How often do you engage in activities that require moderate level of energy such as gardening, cleaning the car, or going for a walk?  Once a week or more often = 0; One to three times a month = 1; Hardly ever, or never = 1 |
