# Supplementary material for: The association between medication or alcohol use and the incidence of frailty: a retrospective cohort study
Source: BMC Geriatr. 2021 Jan 7;21:25. doi: 10.1186/s12877-020-01969-y (PMC7791729; doi:10.1186/s12877-020-01969-y)
Supplement: Supplementary file 2 — Additional file 2: Table B. Variables used to define Frailty index from SHARE data. [file 12877_2020_1969_MOESM2_ESM.docx]

***Additional file 2. Frailty index (FI) defined from SHARE data***

Each participant’s FI score represents the number of age-related deficits as a fraction of the total list of possible age-related deficits. Participants whose data was missing for at least 20% of the complete list of possible deficits were assigned a missing value in the FI outcome. In accordance with previous studies, participants with FI scores ≥ 0.25 were considered to be frail.

**Table B. Variables used to define Frailty index from SHARE data.**

| **Frailty index – 52 items** | **Scoring** |
| --- | --- |
| **General** |  |
| BMI | 0; 0.5; 1 |
| Self-rated health | 0; 0.25; 0.5; 0.75; 1 |
| Hospitalization in past year | 0; 1 |
| Taking medications for chronic conditions | 0; 1 |
| **Comorbidities** |  |
| A heart attack including myocardial infarction or coronary thrombosis or any other heart problem including congestive heart failure | 0; 1 |
| High blood pressure or hypertension | 0; 1 |
| High blood cholesterol | 0; 1 |
| A stroke or cerebral vascular disease | 0; 1 |
| Diabetes or high blood sugar | 0; 1 |
| Chronic lung disease such as chronic bronchitis or emphysema | 0; 1 |
| Cancer or malignant tumor, including leukemia or lymphoma, but excluding minor skin cancers | 0; 1 |
| Stomach or duodenal ulcer, peptic ulcer | 0; 1 |
| Parkinson’s disease | 0; 1 |
| Cataracts | 0; 1 |
| Hip fracture | 0; 1 |
| **Signs and Symptoms** |  |
| Hearing problems | 0; 0.25; 0.5; 0.75; 1 |
| Problems with eyesight - reading | 0; 0.25; 0.5; 0.75; 1 |
| Sleep problems/troubles | 0; 1 |
| **Function** |  |
| Dressing, including putting on shoes and socks | 0; 1 |
| Walking across a room | 0; 1 |
| Bathing or showering | 0; 1 |
| Eating, such as cutting up your food | 0; 1 |
| Getting in or out of bed | 0; 1 |
| Using the toilet, including getting up or down | 0; 1 |
| Using a map to figure out how to get around in a strange place | 0; 1 |
| Preparing a hot meal | 0; 1 |
| Shopping for groceries | 0; 1 |
| Making telephone calls | 0; 1 |
| Taking medications | 0; 1 |
| Doing work around the house or garden | 0; 1 |
| Managing money, such as paying bills and keeping track of expenses | 0; 1 |
| Walking 100 meters | 0; 1 |
| Sitting for about two hours | 0; 1 |
| Getting up from a chair after sitting for long periods | 0; 1 |
| Climbing one flight of stairs without resting | 0; 1 |
| Stooping, kneeling, or crouching | 0; 1 |
| Reaching or extending your arms above shoulder level | 0; 1 |
| Pulling or pushing large objects like a living room chair | 0; 1 |
| Lifting or carrying weights over 10 pounds/5 kilos, like a heavy bag of groceries | 0; 1 |
| Picking up a small coin from a table | 0; 1 |
| Grip strength | 0; 1 |
| **Cognition** |  |
| Delayed recall test | 0; 1 |
| Verbal fluency score | 0; 1 |
| Numeracy – subtraction | 0; 1 |
| **Mental well-being** |  |
| Depression (part of EURO-D) | 0; 1 |
| Pessimism (part of EURO-D) | 0; 1 |
| Suicidality (part of EURO-D) | 0; 1 |
| Interest (part of EURO-D) | 0; 1 |
| Irritability (part of EURO-D) | 0; 1 |
| Appetite (part of EURO-D) | 0; 1 |
| Fatigue (part of EURO-D) | 0; 1 |
| Concentration (part of EURO-D) | 0; 1 |
